# Using droplet digital PCR for the detection of *hco-acr-8b* levamisole resistance marker in *H. contortus*

**DOI:** 10.1016/j.ijpddr.2021.03.002

**Published:** 2021-03-26

**Authors:** Paulius Baltrušis, Claude L. Charvet, Peter Halvarsson, Sofia Mikko, Johan Höglund

**Affiliations:** a Department of Biomedical Sciences and Veterinary Public Health, Section for Parasitology, Swedish University of Agricultural Sciences, Uppsala, Sweden; b ISP, INRA, Université Tours, UMR1282, 37380, Nouzilly, France; c Department of Animal Breeding & Genetics, Swedish University of Agricultural Sciences, Uppsala, Sweden

**Keywords:** ddPCR, *Haemonchus contortus*, Hco-acr-8b, Levamisole, Resistance, Anthelmintic resistance, AR

## Abstract

The nematode *Haemonchus contortus* is one of the most prevalent and pathogenic parasites in small ruminants. Although usually controlled using anthelmintics, the development of drug resistance by the parasite has become a major issue in livestock production. While the molecular detection of benzimidazole resistance in *H. contortus* is well developed, the molecular tools and protocols are far less advanced for the detection of levamisole resistance. The *hco-acr-8* gene encodes a critical acetylcholine susceptible subunit that confers levamisole-sensitivity to the receptor. Here, we report the development of a droplet digital PCR assay as a molecular tool to detect a 63 bp deletion in the *hco-acr-*8 that has been previously associated with levamisole resistance. Sanger sequencing of single adult *H. contortus* yielded 56 high-quality consensus sequences surrounding the region containing the deletion. Based on the sequencing data, new primers and probes were designed and validated with a novel droplet digital PCR assay for the quantification of the deletion containing “resistant” allele in genomic DNA samples. Single adult worms from six phenotypically described isolates (n = 60) and from two Swedish sheep farms (n = 30) where levamisole was effective were tested. Even though a significant difference in genotype frequencies between the resistant and susceptible reference isolates was found (p = 0.01), the homozygous “resistant” genotype was observed to be abundantly present in both the susceptible isolates as well as in some Swedish *H. contortus* samples. Furthermore, field larval culture samples, collected pre- (n = 7) and post- (n = 6) levamisole treatment on seven Swedish sheep farms where levamisole was fully efficacious according to Fecal Egg Count Reduction Test results, were tested to evaluate the frequency of the “resistant” allele in each. Frequencies of the deletion ranged from 35 to 80% in the pre-treatment samples, whereas no amplifiable *H. contortus* genomic DNA was detected in the post-treatment samples. Together, these data reveal relatively high frequencies of the 63 bp deletion in the *hco-acr-8* both on individual *H. contortus* and field larval culture scales, and cast doubt on the utility of the deletion in the *hco-acr-8* as a molecular marker for levamisole resistance detection on sheep farms.

## Highlights

• Acr8b – levamisole resistance marker investigated in single worms and larval cultures.
• Individuals homozygous for acr8b found more commonly, even in susceptible isolates.
• Levamisole treatment efficacy was unaffected by increased acr8b frequencies in larvae.

## Graphical abstract

**Figure:**
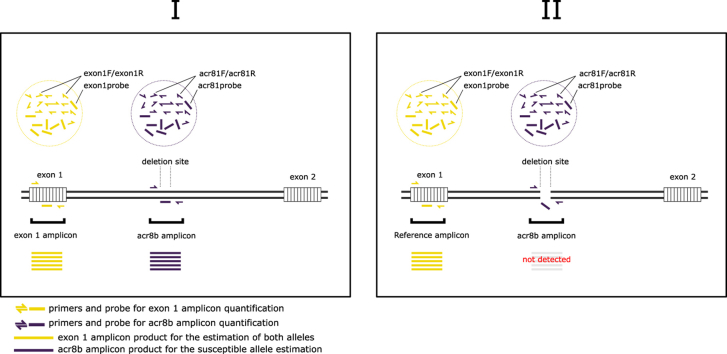

## 1 Introduction

*Haemonchus contortus* is one of the most pathogenic and commonly encountered haematophagous, parasitic gastrointestinal nematode (GIN) species, responsible for contributing to productivity and profitability setbacks in the small-ruminant farming sector across the world (Charlier et al., 2014, 2020). To date, the most effective and reliable measure to control *H. contortus* (as well as other GIN) infections is through the use of broad-range anthelmintic drugs. However, there is a limited number of drugs available and widespread anthelmintic resistance to all major drug classes used to treat sheep has been reported worldwide (Kaplan, 2004; Rose et al., 2015; Kotze and Prichard, 2016).

Levamisole is a broad-spectrum anthelmintic drug, used for the treatment of GIN-infected sheep beginning in the 1960s. It exerts its effect by targeting the nicotinic acetylcholine receptors (nAChRs) present in nematode body-wall muscles, where activation of the receptor by levamisole causes spastic muscle paralysis that, subsequently incapacitates the worm, resulting in its expulsion from the host (Martin et al., 2012). Although generally considered as a last-line drug in terms of efficiency, reports describing levamisole-resistant field *H. contortus* range from multiple-decades old (van Wyk et al., 1989; Waruiru, 1997) to quite recent (Almeida et al., 2010; Chaparro et al., 2017). Nevertheless, the development of levamisole resistance in the field appears to be somewhat slower in comparison to other drug classes (Rose Vineer et al., 2020). Therefore, in some regions, levamisole remains the last efficient solution when the other drugs are no longer effective (Cristel et al., 2017; Kelleher et al., 2020).

Within the context of Sweden, levamisole has only been rarely used (relative to benzimidazoles – BZs and macrocyclic lactones – MLs), and only on farms, where treatment failure with BZ and ML compounds has been confirmed by the Fecal Egg Count Reduction Test (FECRT; Farm and Animal Health Service, www.gardochdjurhalsan.se). Since the number of cases of resistance to the BZ and ML drug classes has been increasingby pathogenic species such as *H. contortus* (Höglund et al., 2009, 2015), the use of levamisole is almost certain to increase with time. Therefore, in an attempt to anticipate the inevitable rise in levamisole resistance in *H. contortus*, the identification of reliable molecular markers associated with resistance and the subsequent development of resistance-detection assays, capable of screening field populations, would be of major interest.

Initial studies, performed in *Caenorhabditis elegans*, identified the role of acetylcholine receptors in mediating levamisole resistance (Lewis et al., 1980), which has prompted further investigation in parasitic nematodes. In *H. contortus*, levamisole resistance was shown to result either from changes in the binding characteristics of the levamisole-sensitive nAChRs (L-AChRs) or in the reduction of the number of channels (Sangster et al., 1988, 1998). More recently, the molecular composition of the *H. contortus* L-AChRs was deciphered (Boulin et al., 2011). A candidate gene approach allowed for the identification of the four subunits (Hco-UNC-29.1, Hco-UNC-38, Hco-UNC-63 and Hco-ACR-8) that make up the L-AChRs (Neveu et al., 2010). Furthermore, the reconstitution of functional L-AChRs required three ancillary proteins and demonstrated the key role of the Hco-acr-8 subunit in the sensitivity to levamisole *in vitro* and *in vivo* (Boulin et al., 2011; Blanchard et al., 2018). However, as pointed out in the review by Kotze and Prichard (2016), evidence seems to suggest at least three different pathways by which resistance to levamisole can develop – truncation of nAChR genes, reduced transcription of nAChR genes and reduced transcription of ancillary protein genes.

Fauvin et al. (2010) described an alternatively spliced transcript for the *hco-acr-8*, which was specifically expressed in three *H. contortus* isolates resistant to levamisole. This finding was subsequently confirmed in other studies using *H. contortus* isolates from different geographical origins (Williamson et al., 2011; Sarai et al., 2013). A few years later, Barrère et al. (2014) not only confirmed the link between the production of the *hco-acr-8b* transcript and levamisole resistance, but also established that the transcript arises due to a 63 bp deletion in the second intronic region. It is worth noting, that, although the deletion was found to be significantly associated with phenotypic resistance in *H. contortus* (p < 0.01), the authors observed that the presence or lack of the 63bp deletion does not account for phenotypic resistance or susceptibility in each and every case. Nevertheless, this led to another recent study (Santos et al., 2019), which looked into the frequency of the truncated, deletion containing *hco-acr-8b* gene in field populations of *H. contortus* in Brazil. The authors of the stated study found a significant positive association (p < 0.05) between the approximated frequency of the resistance-causing (i.e. deletion containing) allele and EC50 as well as EC95 values, determined through larvae development tests, performed on five farm populations. Due to the limited number of samples and sequences, upon which the qPCR assay was based, further investigation is necessary.

There is a clear need to develop molecular tools for levamisole resistance detection (Kotze et al., 2020) and further validate the association between *hco-acr-8b* and levamisole resistance in the field. We have previously successfully utilized droplet digital PCR (ddPCR) to screen for BZ-resistance associated mutations in *H. contortus* (Baltrušis et al., 2018,^2020^). In this study, we have developed and optimized a ddPCR assay to discriminate between the “susceptibility” and “resistance”-associated 63 bp deletion. The primers and probes utilized in our ddPCR assay were designed based on the sequence information retrieved from the second intron region of the *hco-acr-8* (spanning the 63 bp deletion region) in single adult *H. contortus* genomic DNA (gDNA) samples, derived from levamisole-susceptible or -resistant reference isolates as well as from Swedish sheep farms. Our optimized assay was subsequently used for the quantification of the deletion in i) a collection of phenotypically characterized adult worm isolates and ii) adult worm and larval culture samples from farms, wherein levamisole was still effective, according to the FECRT.

## 2 Materials and methods

### 2.1 Sample origin and DNA extraction

Worms of Swedish origin and reference isolates were all previously collected, bio-banked samples of single adult *H. contortus*. As references, we used gDNA from male worms belonging to the levamisole-susceptible isolates - *ISE* (Inbred-susceptible-Edinburgh; henceforth referred to as S1), HcoWEY (Weybridge; S2), HcoZA (Zaïre; S3), and three levamisole-resistant isolates HcoCE (Cedara; R1), HcoRHS6 (Borgsteede; R2), HcoKOK (Kokstad; R3), which all have been phenotypically characterized and described previously (Hoekstra et al., 1997; Fauvin et al., 2010; Neveu et al., 2010; Barrère et al., 2014). The male and female *H. contortus* of Swedish origin were opportunistically collected in the field from the abomasa of culled sheep either by the farmer or a veterinarian. Some of these adult-stage individuals were recovered from animals on two farms (A and B), where levamisole has been proven to be effective, according to the FECRT. Farm A was sampled both in 2018 and 2020, whereas farm B only in 2020.

Paired, pooled larval cultures, each from between 10 and 15 animals (~2g feces/animal), were obtained pre- (n = 7) and 7–10 days post- (n = 6) treatment with levamisole from seven farms around Sweden. These samples were prepared and harvested using the Petri-dish method, as described earlier (Elmahalawy et al., 2018). For farms A, B, C, D, E and F, pre- and post-treatment samples were utilized, whereas for farm G only the pre-treatment sample was available.

gDNA of *H. contortus* single-adult worms or larval cultures of Swedish origin was extracted using the NucleoSpin tissue kit (*Macherey Nagel*, Germany), whereas gDNA from reference isolate worms – was isolated using the DNeasy blood and tissue kit (Qiagen, Germany), according to the manufacturer's recommendations. Finally, either 1 μl (for ddPCR) or 5 μl (for conventional PCR) of the extracted DNA were used in the following analyses.

### 2.2 Fecal egg count reduction test

Two tablespoons of fresh feces were collected from each animal for every tested farm. Generally, 15 sheep were sampled pre-levamisole treatment and 10 sheep with the highest pre-treatment egg counts 7–10 days post-treatment. The fresh individual samples were then immediately placed in separate marked, airtight (zip-locked) plastic bags and shipped overnight by the national post service to the diagnostic laboratory (Vidilab AB). Subsequently, 3 g of feces were screened for strongyle eggs using a modified McMaster method with a minimum diagnostic sensitivity of 50 eggs per gram (EPG) as described earlier (Ljungström et al., 2018).

### 2.3 Amplicon sequencing and primer design

Single worm genomic DNA was used to amplify (AmpliTaq Gold™ DNA Polymerase, ThermoFisher) the partial second intronic region of the *hco-acr-8* using previously suggested primers (“ForInsert” - 5′ACCTTACCTATACACCCGTC3′ and “RevInsert” - 5′CTTGCCGTTATTACACCCTCG3′) and protocol (Barrère et al., 2014). In short, the amplification protocol included a single cycle of 94 °C for 3 min, 40 cycles of 94 °C for 30 s, 55 °C for 30 s, and 68 °C for 30 s, as well as a final extension cycle of 68 °C for 5 min (MyCycler™ Thermal Cycler). Amplicon DNA was quantified (Qubit™ dsDNA HS), cleaned up enzymatically (Exonuclease I [20 U/μL; Thermofisher Scientific] and FastAP Thermosensitive Alkaline Phosphatase [1 U/μL; Thermofisher Scientific], according to the manufacturers guidelines) and submitted for Sanger dideoxy sequencing in both directions to Macrogen Europe.

The obtained sequenced amplicon data was evaluated for quality and trimmed. Upon unsuccessful attempts to split heterozygous indels, consensus sequences were derived from forward and reverse reads (where possible) and aligned using the *CodonCode Aligner* software (v.9.0.1; CodonCode Corporation, Massachusetts, USA). Subsequent evaluation was performed by manually examining the alignments.

The sequenced amplicon data has been deposited to GenBank (accession numbers: MT679733- MT679792).

The assembled sequences were used to manually create a new pair of primers to be used in the ddPCR assay - ACR8F1 (5′CTCCATATTCGAGTTGTGTCTT3′) and ACR8R1 (5′GTATCCAACATTGAATTAAAGGC3′), which create amplicons of either 182 bp (full-length sequence) and/or 119 bp (containing the 63 bp deletion). The optimal annealing temperature was determined via a gradient PCR (Supplementary figure 1). After optimization, the amplification steps for using the pair of primers were as follows: 1 cycle of 95 °C for 5 min, 40 cycles of 95 °C for 45 s, 56 °C for 30 s, 72 °C for 1 min, and a single cycle of 72 °C for 10 min. The products were visualized using GelRed® dye on a 2% agarose gel.

The primers for the reference amplicon in the exon 1 of the *Hco-acr-8* gene were developed *in silico* using the available sequence information for the *hco-acr-8* (HCON_00151270) on the WormBase ParaSite database.

### 2.4 Droplet digital PCR (ddPCR)

#### 2.4.1 Assay setup

The ddPCR assay was designed to use two primer and probe pairs (Fig. 1). The first pair was designed to quantify the presence of the “susceptible” allele, i.e. to estimate the number of amplicon copies/μl for the *hco-acr-8* allele, containing the full-length intronic sequence (forward – “ACR8F1” 5′CTCCATATTCGAGTTGTGTCTT3’; reverse –“ACR8R1” 5′GTATCCAACATTGAATTAAAGGC3’; probe – “ACR8P1” 5’/56-FAM/ATCGCCGCAGTACGCGTAAGGCTGATTA/3IABkFQ/3′). Therefore, in samples, containing the “susceptible” allele (i.e. no deletion is present), the probe will bind and, upon cleavage, produce a fluorescence-emitting molecule, whose signal is measured. However, in samples where the deletion containing “resistant” allele is present, the probe does not bind and, therefore, no detection and quantification measurements are generated. Thus, the ratio of fluorescence forms the basis of the genotyping assay – homozygous susceptible individuals (SS) will emit fluorescence, heterozygous individuals (RS) will generate a fluorescence signal equal to approximately half that produced by the SS, while the homozygous resistant individuals (RR) will not produce any fluorescence. The second primer and probe pair was designed to anneal and estimate the copy number of a short reference amplicon (152 bp) in exon 1 of the *hco-acr-8*.As an independent amplicon, the product should be amplified regardless of whether the downstream intron region contains the deletion or not and, thus, provides a robust reference measurement for the sum of both alleles in a single sample (forward – “Exon1F1” 5′GTCTATGATACGGATAAGCG3’; reverse – “Exon1R1” 5′CAATCGTCGTATACATAGTGG3’; probe – “Exon1P1” 5’/5HEX/CGTTCTTTACCGGTCGCACA/3IABkFQ/3′). By running the primer and probe pairs simultaneously, we established the frequency (%) of the “resistant” allele (*a*) for each sample according to the formula:

$$a=\frac{c-b}{c}\times 100\left(\%\right)$$

Where *b* is the average obtained copy number for the “susceptible” allele and *c* is the average copy number for the reference amplicon in exon 1.

**Fig. 1:**
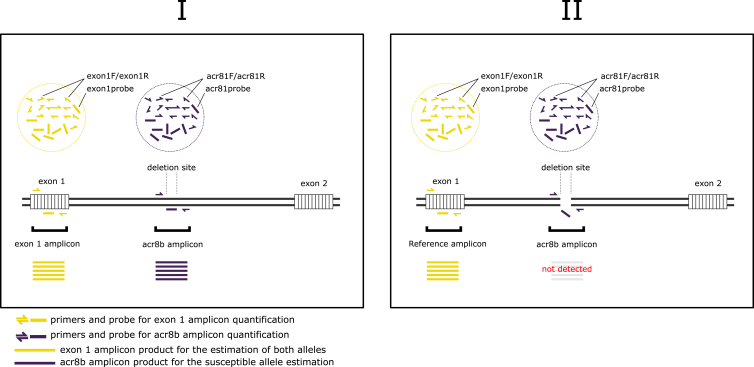
The setup of the ddPCR assay for the estimation of the alleles containing the 63 bp deletion in the *hco-acr-8* in *H. contortus*. (I) and (II) depict the quantification outcomes for both allele types, i.e. when the 63 bp deletion is not present (full-length intron sequence – “susceptible” allele; amplification occurs) and present (amplification occurs, but the probe does not bind, and thus, no detection takes place), respectively. (I) Should the “susceptible” allele be encountered, the amplification of exon 1 and the deletion-containing region occur, resulting in equal amounts of amplicon copies and fluorescence signals, produced by the cleavage of both probes (II) If the “resistant” (i.e. deletion containing). allele is encountered, the fluorescence of only the probe binding to exon 1 is recorded. Primers-probe and exon 1 amplicon are in yellow; Primers/probe and acr8b amplicon are in dark purple; Genomic DNA is in black; Exons 1 and 2 are white, striped rectangles. (For interpretation of the references to colour in this figure legend, the reader is referred to the Web version of this article.)

#### 2.4.2 Assay validation and reaction conditions

The optimal annealing temperature for the primers and probes was determined by performing a gradient ddPCR (Supplementary figure 2). The primer and probe pairs were then used to quantify both amplicons separately, as well as together in a single reaction mix, in order to establish whether or not either of the primer and probe pair mediated amplifications significantly influence the quantification of the other amplicon. To test this, five, individual worm gDNA samples (previously analyzed using Sanger sequencing and with conventional PCR) were evaluated in terms of copy number measurements for both the reference and “sensitive” allele amplicons in *hco-acr-8*. To ensure that technical differences in allele ratios (“susceptible” vs. “resistant”) could be reliably estimated in a mixed population setting, a linear association between the dilution factor and the frequency of the “resistant” allele was estimated by mixing the gDNA derived from a phenotypically resistant isolate sample (Cedara3) with gDNA from a previously sequenced susceptible (i.e. not containing the 63bp deletion) Swedish field isolate sampled at various ratios of the two DNA pools (100:0, 80:20, 60:40, 50:50, 40:60, 20:80 and 0:100).

The sample reactions were assembled in 96-well plates (final volume 22 μl), following the guidelines issued by the manufacturer (BioRad). Droplets were generated and dispensed into a new 96-well plate using an automated droplet generator (QX200, BioRad). The new plate was heat sealed and transferred into a thermal cycler (MyCycler™ Thermal Cycler). The optimized PCR conditions were as follows: a single cycle of 95 °C for 10 min, 40 cycles of 94 °C for 30 s and then 58 °C for 1 min, followed by a final cycle of 98 °C for 10 min to deactivate the enzyme. After the amplification step, the plate containing the droplets was loaded into the droplet reader (QX200, BioRad) and further analyzed using QuantaSoft (v1.April 7, 0917) software, which generates DNA copy measurements, fractional abundance data, and error bars based on Poisson statistics (Hindson et al., 2011). No-template control samples were included in every run to monitor for possible contamination.

#### 2.4.3 Analysis

The output from QuantaSoft was visualized using the *ggplot2* package (v3.2.1) for R software (v3.6.3). The frequencies of the genotypes for reference isolates (SS; RS; RR) were analyzed with Pearson's Chi-squared test in R (v3.6.3).

## 3 Results

### 3.1 Amplification and sequencing

To design primers and probes to assay the 63 bp deletion site in the *hco-acr-*8, we first amplified and sequenced the second intron region containing the 63 bp deletion site in the *hco-acr-8* from 90 samples of individual adult *H. contortus* (42 of Swedish origin, 48 – reference isolates) using the previously described PCR protocol by Barrère et al. (2014). Among these, 56 individual samples (21 of Swedish origin, 35 - reference isolates) produced high-quality, full-sequence length sequences and were retained (Supplementary figure 3), whereas 34 samples yielded either only partially resolved or were low-quality, i.e. noisy (especially surrounding the area of the deletion), and were removed from further analysis. No heterozygous individuals were found upon attempting to ‘split heterozygotes indels’ within the *CodonCode Aligner* software and, therefore, only the major allele was recovered for each sample. Out of the 56 sequences, 14 (25%) did not possess the deletion (11 sequences of Swedish origin and 3 - reference isolates), whereas the other 42 (75%) presented the expected 63 bp deletion (10 of Swedish origin, 32 - reference isolates).

The reference isolate samples were further genotyped using primers ACR8F1 and ACR8R1 (10 worms per isolate: ISE, Weybridge, Zaïre, Cedara, Borgsteede, Kokstad) (Supplementary figures 4 and 5). Out of the 35 sequenced reference isolate samples, four individuals (11%) were incorrectly genotyped as RR by sequencing, where further analysis with conventional PCR found these to be RS (Supplementary table 1). The genotypes of the remaining worms were concordant between the two independent assays.

### 3.2 Droplet digital PCR

#### 3.2.1 Adult worms

The newly developed droplet digital PCR approach was first evaluated for potential cross-reactivity between the two primer and probe sets in a single reaction mix as well as for the technical consistency of the “susceptible” and “resistant” allele frequency estimation via increasing dilutions.

Optimally, both primer and probe pairs for the detection of the target and reference amplicons would be used in the same reaction. Due to the possibility of primer and/or probe interactions (i.e. cross-reactivity) resulting in a reduced efficiency of the assay, we tested for this bias by performing both amplicon quantifications in single-plex (one primer and probe pair) and duplex reactions (both primer and probe pairs) for comparison. Quantifications of the amplicons were not influenced by the presence of the other primer and probe pair in the mix, as measurements between the single-plex and duplex reactions were highly similar across the five individual gDNA samples (Fig. 2). Moreover, the previously established genotypes (Fig. 2B) for these five samples were confirmed with our ddPCR assay – adult1 and adult2 were SS, adult3-RS, whereas adult4 and adult5 were of RR genotype. Linear increase in the ratio of the “resistant” allele was observed as the proportion of “susceptible” allele containing gDNA was reduced through increasing dilutions (Spearman correlation = 0.9975; Supplementary figure 6).

**Fig. 2:**
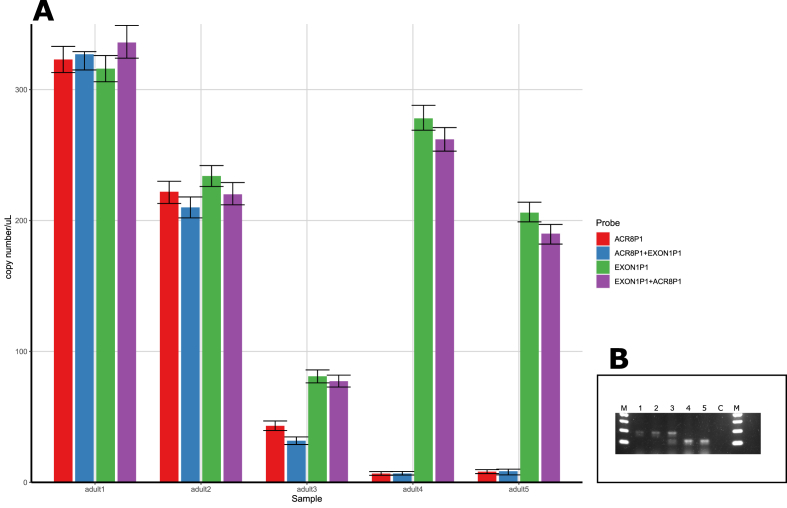
(A) Cross-reactivity examination between primers and probe sets for the quantification of the 63 bp deletion as well as exon 1 amplicon in five adult *H. contortus* of distinct genotypes (two homozygous “susceptible” (SS), one heterozygous (RS) and two homozygous “resistant” (RR)). Amplicon DNA copy number was estimated for the “susceptible” full-length *hco-acr-8* fragment as well as exon 1 (in separate fluorescence detection channels) using individual primer and probe sets (“ACR8P1” and “EXON1P1” in the figure) and both sets together (“ACR8P1+EXON1P1”, “EXON1P1+ACR8P1” in the figure). In red: DNA copy number for the “susceptible”, full-length *hco-acr-8*, obtained by analyzing the specific primers and probe in a single-plex reaction setup. In blue: DNA copy number for the “susceptible”, full-length *hco-acr-8*, obtained by analyzing both primer and probe sets in a single duplex reaction. In green: DNA copy number for the amplicon in exon 1, obtained by analyzing the specific primers and probe in a single-plex reaction setup. In purple: DNA copy number for the amplicon in exon 1, obtained by analyzing both primer and probe sets in a single duplex reaction. Error bars represent 95% Poisson confidence interval values. (B) shows the genotype of each individual (adults (1–5), as confirmed by conventional PCR using primers ACR8F1 and ACR8R1. M – 100bp DNA ladder (ThermoFisher Scientific). C – negative template control. (For interpretation of the references to colour in this figure legend, the reader is referred to the Web version of this article.)

**Fig. 3:**
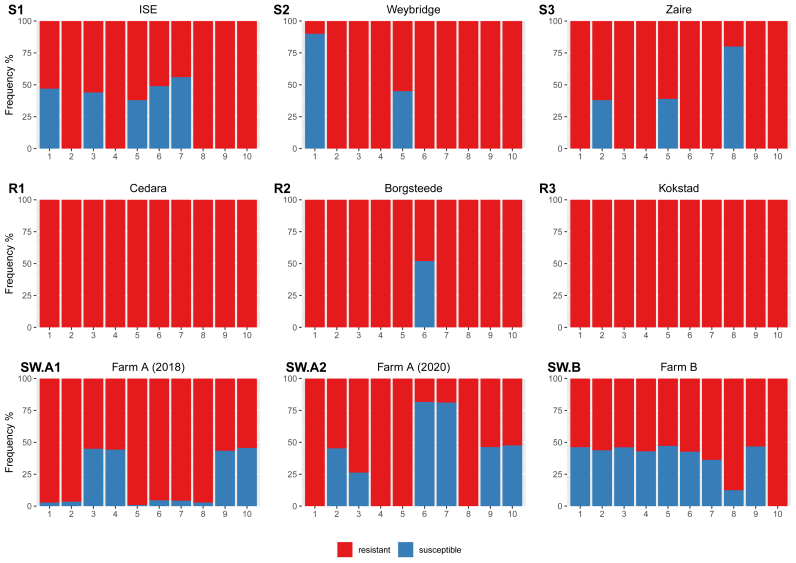
The frequencies (%) of “susceptible” (full-length second intron sequence; in blue) and “resistant” (63 bp deletion in the second intron in the *hco-acr-8*; in red) alleles in various individual, adult *H. contortus* isolates. S1–S3 correspond to levamisole susceptible isolates – ISE, Weybridge and Zaïre, whereas R1-R3 to levamisole resistant – Cedara, Borgsteede and Kokstad. SW.A1, SW.A2 and SW.B represent allele frequencies in individual, adult *H. contortus* isolated from farms A (in 2018 and then 2020) and B, respectively. (For interpretation of the references to colour in this figure legend, the reader is referred to the Web version of this article.)

The previously examined, reference isolate genomic DNA samples as well as single, adult worms derived from two Swedish farms (wherein levamisole was determined to be efficacious according to the FECRT; Table 1) were evaluated with the ddPCR assay in order to estimate the frequencies of the “resistant” allele (Fig. 3). After the comparison of reference isolate genotype data (obtained with both conventional and ddPCR) and due to the observed difference in amplification efficiency between the two primer-probe sets, cut-off values were set for SS- (<25% frequency of the “resistant” allele), RS- (between 25 and 75% frequency of the “resistant” allele) and RR- (>75% frequency of the “resistant” allele) genotype-possessing individuals.

**Table 1:** Strongylid eggs per gram of feces counts obtained from sheep (n = 10–15) on farms (A-G), from which the before and after (7–10 days) levamisole treatment, *H. contortus*-dominated, field larvae populations were derived.

Farm	Average pre-treatment EPG count	Standard deviation	Average post-treatment EPG count	Efficacy of treatment (%)
A	6340	4510	0	100
B	5356	3787	0	100
C	394	308	0	100
D	6340	4510	0	100
E	419	827	0	100
F	195	152	0	100
G	292	228	NOT TESTED	–

Among the 60 ddPCR-genotyped reference isolates, five (8%; samples ISE1,3,5,6,7) were found to be RS, instead of either RR or SS (as determined previously by conventional PCR). Furthermore, in comparison to the sequencing data, four samples (6%; ISE5, Zaire2, Zaire5, Borgsteede6) were identified as RS, instead of either RR or SS (Supplementary table 1).

In total, eight RS and two SS samples were identified within the phenotypically susceptible isolate category (S1–S3; n = 30), whilst all but one worm (Borgsteede6 - RS) were found to be RR in the phenotypically resistant isolate group (R1-R3; n = 30) (Fig. 3). Although Pearson's Chi-squared test showed significant difference (p = 0.01) in the genotype (SS, RS, RR) frequencies between the susceptible and resistant isolates, the RR genotype was the most common in both categories, irrespective of phenotypic status.

In addition, 30 individual adult worm gDNA samples from the two Swedish farms (SW.A1, SW.A2 and SW.B; Fig. 3) were tested for the frequencies of the “resistant” allele. Overall, 16 gDNA samples were deemed to be of RS (the most common genotype) and 12 of RR genotype, whilst only two were found to be SS.

#### 3.2.2 Larval cultures

The pre-treatment FECs varied between 195 ± 152 (on farm F) to 6340 ± 4510 EPG (on farm A), whereas all post-treatment samples were negative. The reduction on all six farms was 100% (Table 1).

**Fig. 4:**
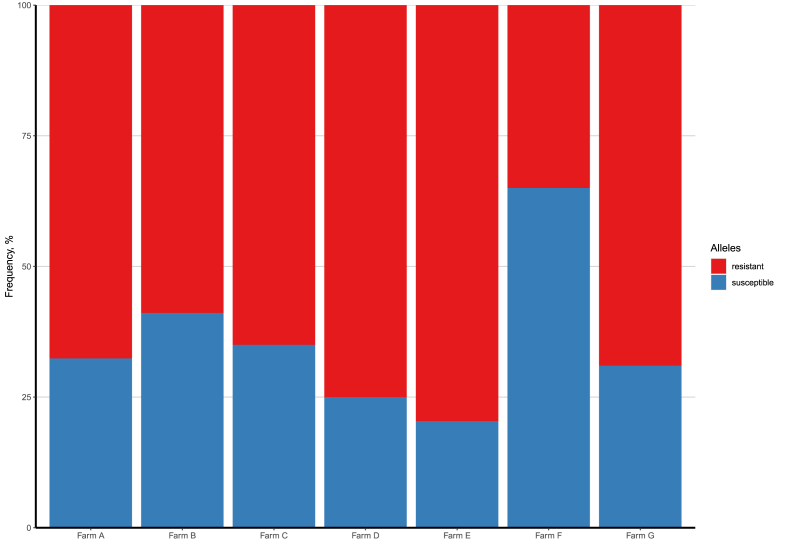
The frequencies (%) of “susceptible” (full-length second intron sequence; in blue) and “resistant” (63 bp deletion in the second intron of the *hco-acr-8*; in red) alleles in larvae cultures, recovered from different farms (A, B, C, D, E, F, G) pre-treatment with levamisole. Note: All six post-treatment samples did not contain *H.contortus* DNA (thus are not shown) whereas population G did not have a post-treatment sample taken, due to low egg counts in the pre-treatment samples. (For interpretation of the references to colour in this figure legend, the reader is referred to the Web version of this article.)

The ddPCR assay was subsequently used to screen all six paired (A-F) and one unpaired (G) field-derived larval culture samples (producing on average a total of 11,5–9717 copies/μl) (Fig. 4). The “resistant” allele was detected in all pre-treatment larvae culture samples at ratios ranging 35–80%. In contrast, all post-treatment samples contained no trace of *H. contortus* amplicon DNA (data not shown).

## 4 Discussion

Due to the worrying state of highly pathogenic GINs, such as *H. contortus*, found exhibiting elevated resistance to benzimidazoles, macrocyclic lactones and monepantel (Höglund et al., 2009, 2015,2020), it is vitally important to maintain the efficacy of levamisole – currently the “last line of defense” drug against GIN infections in small ruminants in Sweden. In order to monitor the development of levamisole resistance by *H. contortus* on Swedish sheep farms, a molecular screening tool for the rapid detection and estimation of resistance-encoding alleles in field samples would be of immense use. Here, we have described the development of an assay to detect a 63 bp deletion in the *hco-acr-8* L-AChR subunit gene (Barrère et al., 2014) previously identified to be associated with levamisole resistance.

Using the obtained sequencing data for intron 2 amplicon in *hco-acr-8* from 56 adult *H. contortus*, a primer pair was developed to be used in further genotyping (with conventional PCR and ddPCR) of individual worm gDNA samples. Although amplification was observed for all samples, varying degrees of amplification efficiency were found not only between the different individuals but also between the two alleles belonging to the same worm (Supplementary figures 4 and 5). This is in agreement with previous work (Barrère et al., 2014) and possibly explains why only the major allele was possible to retrieve from each sequenced single worm sample. Whilst DNA concentration could have played a role, considering the normally high mutation frequencies within the intronic regions in eukaryotic organisms, this can most likely be attributed to nucleotide variation within the primer binding sites.

Despite the limitations in identifying RS genotypes with both Sanger sequencing as well as conventional PCR, the results for the comparisons between the direct deletion detection (sequencing and conventional PCR) and indirect deletion detection (ddPCR) assays in terms of identifying the genotypic status of each of the tested reference *H. contortus* isolate are overall congruent (Supplementary table 1). However, the clear advantage of ddPCR over conventional PCR is that the former is claimed to eliminate amplification bias by constraining the results of the amplification to a binary outcome (Hindson et al., 2011). In addition, by validating our ddPCR assay, we not only established that both primer and probe pairs do not cross-react with one another when used in a duplex reaction (Fig. 2) but also that the dilution of the gDNA containing only the “susceptible” allele with gDNA containing only the “resistant” allele results in a perfect linear pattern of decrease in the frequency of the diluted allele (and an increase in the “resistant” allele; Supplementary figure 6). However, it is important to point out that cross-reactivity between the primers and probes in a single reaction is fundamentally different from biases, resulting from variation in primer and probe binding efficiencies. Furthermore, the calculated correlation between the sample dilution and allele frequency reflects only the technical variability and the capacity of the assay to distinguish and estimate different proportions of the “sensitive” and reference allele amplicons in a sample. Thus, while a good linear relationship across the dilution series was observed (Supplementary figure 6), the accuracy is not as good. Samples, containing only the “sensitive” allele did not reach a 100% in the frequency of this allele (or 50% in the case of RS genotypes), likely due to differences in amplification efficiency between the two amplicons. Fundamentally, ddPCR estimates the intensity of fluorescence produced by the amplification of a distinct amplicon (and converts that intensity into target copies/μl) and, therefore, the subsequent copy measurements depend not only on the DNA concentration in the sample, but also the degree of nucleotide variability. Therefore, we believe this inconsistency between the copy numbers for two amplicons to be a direct consequence of the reference amplicon being situated in a more conserved exonic region, whereas the “sensitive” allele amplicon was in an inherently more variable intron. Nevertheless, unlike in any of our previous approaches for the detection of SNPs (Baltrušis et al., 2018) or genetically variable regions for species differentiation (Baltrušis et al., 2019), this study employs a more robust design, utilizing the simultaneous absolute quantification of two distant regions within the *hco-acr-8* for the indirect determination of the frequency of the “resistant” allele.

Having examined the six reference isolates (n = 10 per isolate) using the ddPCR platform, it was found that the genotypic status of individuals within the reference isolate groups (S1–S3 and R1-R3) agreed for the most part with the results obtained with conventional PCR. Notably, in the case of S1 (ISE isolate) which has been previously confirmed to be of RS genotype for the 63bp deletion (Barrère et al., 2014), conventional PCR failed to elucidate the heterozygosity due to poor amplification (Supplementary figure 4). However, 50% of worms (5/10) within this isolate were indeed found to be RS when analyzed with the more sensitive ddPCR assay (Fig. 3). Even though ddPCR seemed to provide more sensitive measurements, the “susceptible” allele was, in many cases, underestimated, leading to the overestimation of the “resistant” allele. Thus, neither the 50% frequency of the “susceptible” allele, in the case of RS individuals (i.e. ISE-1,3,5, Weybridge-5 and S3-2,5), nor the 100% for fully SS individuals (i.e. Weybridge-1 and Zaire-8) were reached. The frequency between 25 and 75% of the “susceptible” allele in a single worm sample was, therefore, considered to be indicative of the RS genotype and >75% of SS genotype. A similar pattern of underestimation was also observed for adult worms derived from the Swedish farms (one of which was sampled at two different time points – 2018 and then again in 2020). Here, samples SW.B-8 and SW.A2-3 were severely underestimated in terms of the “susceptible” allele, but were distinguishable from what we considered to be minor contaminations, observed in SW.A1-1, 2, 5, 6, 7 and 8. Quite unexpectedly, individuals possessing the SS genotype were the rarest, even among the phenotypically susceptible isolates (2/30). Moreover, apart from populations SW.B and SW.A2, the “resistant” allele was much more common, even in the reference isolates, confirmed to be susceptible to levamisole (S1–S3). Despite the fact that the difference in the three genotypes (i.e. SS, SR, RR) between the susceptible (S1–S3) and resistant (R1-R3) isolates was statistically significant (p = 0.01), which is in line with Barrère et al. (2014), in well-characterized (and susceptible to levamisole), isolates, such as ISE, Weybridge and Zaïre, half or more of the individuals were found to be of RR genotype. Thus, judging by the obtained genotypes for all six isolates, it appears as the 63bp deletion in *hco-acr-8* is a poor predictor of the actual phenotype in individual worms.

Larvae culture samples, derived from sheep before and after treatment with levamisole, were used to quantify the presence of the deletion in the field (Fig. 4). Not only was the 63bp deletion found in all tested populations (Farms A-G) before treatment, but the frequency of the deletion varied to a great extent (35–80%). Interestingly, in six of those populations no trace of amplifiable *H. contortus* DNA was found in any of the post-treatment samples, indicating that the levamisole treatment had been successful. This observation was consistent with the FECRT data (Table 1). Although it has been previously posited that resistance caused by the deletion is likely to be genetically recessive, i.e. RS individuals might still be susceptible (Santos et al., 2019), overall it appears that the increased frequencies of the deletion in the *hco-acr-8* did not at all correspond to heightened resistance to the effect of levamisole in the field isolates upon treatment. Furthermore, unlike the previously discussed study (Santos et al., 2019), wherein the “resistant” allele frequencies were correlated with LD50 values, the variation in the frequency of the “resistant” allele among the tested Swedish single worm isolates and larval cultures (derived from farms wherein levamisole was efficacious) proved to be somewhat random.

Judging from the results obtained for single worm and larvae culture samples, we conclude that the ddPCR assay cannot be reliably used for the estimation of the deletion frequency in larvae pools. While certain limitations, present for single worm gDNA samples (mainly higher overall efficiency of amplification of the reference allele in comparison to the “sensitive” allele, leading to the overestimation of the frequency of the deletion) can be mitigated by employing cut-off thresholds to define genotypes, the amplification efficiency bias cannot be easily addressed in pools of multiple individuals.

From a practical standpoint, our results demonstrate the 63 bp deletion is not a predictive marker for levamisole resistance status determination in a field population context, as it is apparent that the deletion is not associated with the resistant phenotype in every case. A similar conclusion was drawn by Chagas et al. (2016), who observed the presence of the 63 bp deletion in individual larvae belonging to a susceptible *H. contortus* strain and, thus, determined that the absence of the 63 bp fragment could not always be linked to levamisole resistance in *H. contortus*.

In addition to *hco-acr-8*, other genes of subunits within the L-AChRs, such as *hco-unc-63* (Neveu et al., 2010; Boulin et al., 2011), as well differences in the expression patterns of the auxiliary and P-glycoprotein genes (Sarai et al., 2014), are involved in the determination of the phenotypic status and, thus, could be important contributing forces in the development of resistance. Sarai et al. (2014 and 2015) have demonstrated that not only does the expression of putative candidate genes, thought to be involved in the development of resistance to levamisole, vary considerably depending on the concentration of levamisole administered to the infected animals, but that the expression of those genes (including *hco-acr-8b*) appeared to also fluctuate in different life cycle stages of *H. contortus* (L1, L3 and adults). Interestingly, the latter study also found the adult stages to be persistently susceptible according to the drench efficacy test results (FECRT), even after nine generations of selective propagation and extreme, subsequent increases in resistance, as measured by the larval development assays. Thus, the determination of which key cellular changes in *H. contortus* are essential in order to develop resistance to levamisole as well as to what degree each of these changes contribute to the phenotypic differences in field isolates, remains somewhat elusive.

A significant challenge in this study is related to the heightened nucleotide variation within the intron region where the 63 bp deletion was first discovered. This can adversely affect the precision and overall applicability of the quantitative, diagnostic measurements via biased primer binding and uneven allele amplification, especially when the species under the investigation, such as *H. contortus*, is exceptionally genetically diverse (Yin et al., 2013; Sallé et al., 2019; Doyle et al., 2020). Yet another disadvantage of the current approach is that this assay does not directly detect and estimate the proportion of the deletion containing allele. The alternative approach would be to design a probe, flanking the deleted region, thus, resulting in detection and quantification of only the truncated form. Yet another approach would consist of using degenerate primers and probes. However, such approach would require a more in-depth knowledge of the nucleotide variation present within the deletion surrounding region. Although the current assay setup was chosen to mitigate the observed nucleotide variation, surrounding the 63 bp intronic deletion based on Sanger sequencing, overall, the data presented here, suggests that genetically variable sites, such as intronic regions, are not an ideal target in creating PCR based amplification assays for anthelmintic marker detection and quantification.

In conclusion, we have further evaluated the suitability of the 63 bp deletion in the *hco-acr-8* as a potential marker to track levamisole resistance in *H. contortus* field isolates. The presence of the deletion (i.e. “resistant” allele) in reference isolates as well as Swedish single adult worms and field populations was identified by sequencing, conventional and droplet digital PCRs. However, neither reduced levamisole efficacy, nor an increased proportion of surviving individuals in the field populations, subjected to levamisole treatment were observed, despite the high frequency of the “resistant” allele. Furthermore, although a significant difference (p = 0.01) was found between the reference isolates (susceptible vs. resistant) in terms of the genotype frequencies, the deletion containing “resistant” allele was more prominent, even in the susceptible isolate category. Although ddPCR proved to be a valid molecular tool for mutation detection and quantification (despite some inherent challenges with the current approach), it is important to keep in mind that resistance to levamisole has been reported to be a polygenic trait (Kotze and Prichard, 2016), associated with multiple, perhaps even simultaneous, cellular changes. Thus, further genome-wide approaches, as discussed previously by (Gilleard, 2006) and Doyle and Cotton (2019) are necessary to provide new insights into potentially better molecular markers for levamisole resistance detection in *H. contortus*.

## Declaration of competing interest

The authors have no conflicts of interest to declare.

## Figures and Tables

**figs1:**
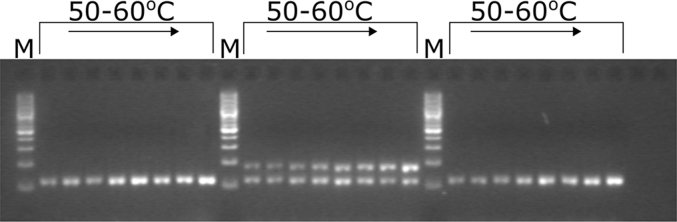


**figs2:**
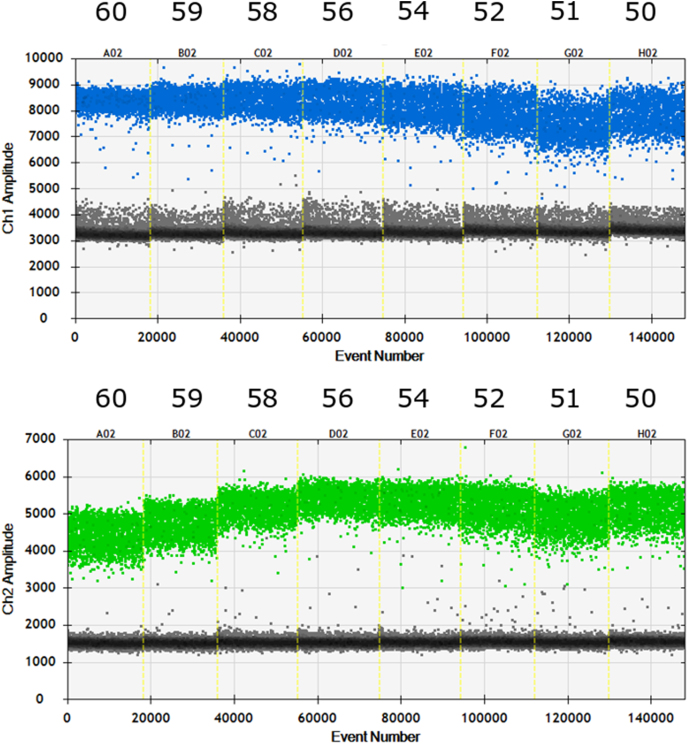


## References

[bib1] Almeida F.A., Garcia K.C.O.D., Torgerson P.R., Amarante A.F.T. (2010). Multiple resistance to anthelmintics by Haemonchus contortus and Trichostrongylus colubriformis in sheep in Brazil. Parasitol. Int..

[bib2] Baltrušis P., Halvarsson P., Höglund J. (2020). Utilization of droplet digital PCR to survey resistance associated polymorphisms in the β tubulin gene of Haemonchus contortus in sheep flocks in Sweden. Vet. Parasitol..

[bib3] Baltrušis P., Halvarsson P., Höglund J. (2019). Molecular detection of two major gastrointestinal parasite genera in cattle using a novel droplet digital PCR approach. Parasitol. Res..

[bib4] Baltrušis P., Halvarsson P., Höglund J. (2018). Exploring benzimidazole resistance in Haemonchus contortus by next generation sequencing and droplet digital PCR. Int. J. Parasitol. Drugs Drug Resist..

[bib5] Barrère V., Beech R.N., Charvet C.L., Prichard R.K. (2014). Novel assay for the detection and monitoring of levamisole resistance in Haemonchus contortus. Int. J. Parasitol..

[bib6] Blanchard A., Guégnard F., Charvet C.L., Crisford A., Courtot E., Sauvé C., Harmache A., Duguet T., O'Connor V., Castagnone-Sereno P., Reaves B., Wolstenholme A.J., Beech R.N., Holden-Dye L., Neveu C. (2018). Deciphering the molecular determinants of cholinergic anthelmintic sensitivity in nematodes: when novel functional validation approaches highlight major differences between the model Caenorhabditis elegans and parasitic species. PLoS Pathog..

[bib7] Boulin T., Fauvin A., Charvet C.L., Cortet J., Cabaret J., Bessereau J.L., Neveu C. (2011). Functional reconstitution of Haemonchus contortus acetylcholine receptors in Xenopus oocytes provides mechanistic insights into levamisole resistance. Br. J. Pharmacol..

[bib8] Chagas A.C. de S., Domingues L.F., Gaínza Y.A., Barioni-Júnior W., Esteves S.N., Niciura S.C.M. (2016). Target selected treatment with levamisole to control the development of anthelmintic resistance in a sheep flock. Parasitol. Res..

[bib9] Chaparro J.J., Villar D., Zapata J.D., López S., Howell S.B., López A., Storey B.E. (2017). Multi-drug resistant Haemonchus contortus in a sheep flock in Antioquia, Colombia. Vet. Parasitol. Reg. Stud. Reports.

[bib10] Charlier J., Rinaldi L., Musella V., Ploeger H.W., Chartier C., Vineer H.R., Hinney B., von Samson-Himmelstjerna G., Băcescu B., Mickiewicz M., Mateus T.L., Martinez-Valladares M., Quealy S., Azaizeh H., Sekovska B., Akkari H., Petkevicius S., Hektoen L., Höglund J., Morgan E.R., Bartley D.J., Claerebout E. (2020). Initial assessment of the economic burden of major parasitic helminth infections to the ruminant livestock industry in Europe. Prev. Vet. Med..

[bib11] Charlier J., van der Voort M., Kenyon F., Skuce P., Vercruysse J. (2014). Chasing helminths and their economic impact on farmed ruminants. Trends Parasitol..

[bib12] Cristel S., Fiel C., Anziani O., Descarga C., Cetrá B., Romero J., Fernández S., Entrocasso C., Lloberas M., Medus D., Steffan P. (2017). Anthelmintic resistance in grazing beef cattle in central and northeastern areas of Argentina — an update. Vet. Parasitol. Reg. Stud. Reports.

[bib13] Doyle S.R., Cotton J.A. (2019). Genome-wide approaches to investigate anthelmintic resistance. Trends Parasitol..

[bib14] Doyle S.R., Tracey A., Laing R., Holroyd N., Bartley D., Bazant W., Beasley H., Beech R., Britton C., Brooks K., Chaudhry U., Maitland K., Martinelli A., Noonan J.D., Paulini M., Quail M.A., Redman E., Rodgers F.H., Sallé G., Shabbir M.Z., Sankaranarayanan G., Wit J., Howe K.L., Sargison N., Devaney E., Berriman M., Gilleard J.S., Cotton J.A. (2020). Genomic and transcriptomic variation defines the chromosome-scale assembly of Haemonchus contortus, a model gastrointestinal worm. Commun. Biol..

[bib15] Elmahalawy S.T., Halvarsson P., Skarin M., Höglund J. (2018). Droplet digital polymerase chain reaction (ddPCR) as a novel method for absolute quantification of major gastrointestinal nematodes in sheep. Vet. Parasitol..

[bib16] Fauvin A., Charvet C., Issouf M., Cortet J., Cabaret J., Neveu C. (2010). cDNA-AFLP analysis in levamisole-resistant Haemonchus contortus reveals alternative splicing in a nicotinic acetylcholine receptor subunit. Mol. Biochem. Parasitol..

[bib17] Gilleard J.S. (2006). Understanding anthelmintic resistance: the need for genomics and genetics. Int. J. Parasitol..

[bib18] Hindson B.J., Ness K.D., Masquelier D.A., Belgrader P., Heredia N.J., Makarewicz A.J., Bright I.J., Lucero M.Y., Hiddessen A.L., Legler T.C., Kitano T.K., Hodel M.R., Petersen J.F., Wyatt P.W., Steenblock E.R., Shah P.H., Bousse L.J., Troup C.B., Mellen J.C., Wittmann D.K., Erndt N.G., Cauley T.H., Koehler R.T., So A.P., Dube S., Rose K.A., Montesclaros L., Wang S., Stumbo D.P., Hodges S.P., Romine S., Milanovich F.P., White H.E., Regan J.F., Karlin-Neumann G.A., Hindson C.M., Saxonov S., Colston B.W. (2011). High-throughput droplet digital PCR system for absolute quantitation of DNA copy number. Anal. Chem..

[bib19] Hoekstra R., Borgsteede F.H.M., Boersema J.H., Roos M.H. (1997). Selection for high levamisole resistance in Haemonchus contortus monitored with an egg-hatch assay. Int. J. Parasitol..

[bib20] Höglund J., Enweji N., Gustafsson K. (2020). First case of monepantel resistant nematodes of sheep in Sweden. Vet. Parasitol. Reg. Stud. Reports.

[bib21] Höglund J., Gustafsson K., Ljungström B.L., Engström A., Donnan A., Skuce P. (2009). Anthelmintic resistance in Swedish sheep flocks based on a comparison of the results from the faecal egg count reduction test and resistant allele frequencies of the β-tubulin gene. Vet. Parasitol..

[bib22] Höglund J., Gustafsson K., Ljungström B.L., Skarin M., Varady M., Engström F. (2015). Failure of ivermectin treatment in Haemonchus contortus infected-Swedish sheep flocks. Vet. Parasitol. Reg. Stud. Reports.

[bib23] Kaplan R.M. (2004). Drug resistance in nematodes of veterinary importance: a status report. Trends Parasitol..

[bib24] Kelleher A.C., Good B., De Waal T., Keane O.M. (2020). Anthelmintic resistance among gastrointestinal nematodes of cattle on dairy calf to beef farms in Ireland. Ir. Vet. J..

[bib25] Kotze A.C., Gilleard J.S., Doyle S.R., Prichard R.K. (2020). Challenges and opportunities for the adoption of molecular diagnostics for anthelmintic resistance. Int. J. Parasitol. Drugs Drug Resist..

[bib26] Kotze A.C., Prichard R.K. (2016). Anthelmintic resistance in Haemonchus contortus: history, mechanisms and diagnosis. Adv. Parasitol..

[bib27] Lewis J.A., Wu C.H., Levine J.H., Berg H. (1980). Levamisole-resitant mutants of the nematode Caenorhabditis elegans appear to lack pharmacological acetylcholine receptors. Neuroscience.

[bib28] Ljungström S., Melville L., Skuce P.J., Höglund J. (2018). Comparison of four diagnostic methods for detection and relative quantification of Haemonchus contortus eggs in feces samples. Front. Vet. Sci..

[bib29] Martin R.J., Robertson A.P., Buxton S.K., Beech R.N., Charvet C.L., Neveu C. (2012). Levamisole receptors: a second awakening. Trends Parasitol..

[bib30] Neveu C., Charvet C.L., Fauvin A., Cortet J., Beech R.N., Cabaret J. (2010). Genetic diversity of levamisole receptor subunits in parasitic nematode species and abbreviated transcripts associated with resistance. Pharmacogenetics Genom..

[bib31] Rose H., Rinaldi L., Bosco A., Mavrot F., de Waal T., Skuce P., Charlier J., Torgerson P.R., Hertzberg H., Hendrickx G., Vercruysse J., Morgan E.R. (2015). Widespread anthelmintic resistance in European farmed ruminants: a systematic review. Vet. Rec..

[bib32] Rose Vineer H., Morgan E.R., Hertzberg H., Bartley D.J., Bosco A., Charlier J., Chartier C., Claerebout E., de Waal T., Hendrickx G., Hinney B., Höglund J., Ježek J., Kašný M., Keane O.M., Martínez-Valladares M., Mateus T.L., McIntyre J., Mickiewicz M., Munoz A.M., Phythian C.J., Ploeger H.W., Rataj A.V., Skuce P.J., Simin S., Sotiraki S., Spinu M., Stuen S., Thamsborg S.M., Vadlejch J., Varady M., von Samson-Himmelstjerna G., Rinaldi L. (2020). Increasing importance of anthelmintic resistance in European livestock: creation and meta-analysis of an open database. Parasite.

[bib33] Sallé G., Doyle S.R., Cortet J., Cabaret J., Berriman M., Holroyd N., Cotton J.A. (2019). The global diversity of Haemonchus contortus is shaped by human intervention and climate. Nat. Commun..

[bib34] Sangster N.C., Riley F.L., Collins G.H. (1988). Investigation of the mechanism of levamisole resistance in trichostrongylid nematodes of sheep. Int. J. Parasitol..

[bib35] Sangster N.C., Riley F.L., Wiley L.J. (1998). Binding of [3H]m-aminolevamisole to receptors in levamisole- susceptible and -resistant Haemonchus contortus. Int. J. Parasitol..

[bib36] dos Santos J.M.L., Vasconcelos J.F., Frota G.A., Freitas E.P. de, Teixeira M., Vieira L. da S., Bevilaqua C.M.L., Monteiro J.P. (2019). Quantitative molecular diagnosis of levamisole resistance in populations of Haemonchus contortus. Exp. Parasitol..

[bib37] Sarai R.S., Kopp S.R., Coleman G.T., Kotze A.C. (2014). Drug-efflux and target-site gene expression patterns in Haemonchus contortus larvae able to survive increasing concentrations of levamisole in vitro. Int. J. Parasitol. Drugs Drug Resist..

[bib38] Sarai R.S., Kopp S.R., Coleman G.T., Kotze A.C. (2013). Acetylcholine receptor subunit and P-glycoprotein transcription patterns in levamisole-susceptible and -resistant Haemonchus contortus. Int. J. Parasitol. Drugs Drug Resist..

[bib39] Sarai R.S., Kopp S.R., Knox M.R., Coleman G.T., Kotze A.C. (2015). In vitro levamisole selection pressure on larval stages of Haemonchus contortus over nine generations gives rise to drug resistance and target site gene expression changes specific to the early larval stages only. Vet. Parasitol..

[bib40] van Wyk J.A., van Schalkwyk P.C., Gerger H.M., Visser E.L., Alves R.M., van Schalkwyk L. (1989). South African field strains of Haemonchus contortus resistant to the levamisole/morantel group of anthelmintics. Onderstepoort J. Vet. Res..

[bib41] Waruiru R.M. (1997). Efficacy of closantel, albendazole and levamisole on an ivermectin resistant strain of Haemonchus contortus in sheep. Vet. Parasitol..

[bib42] Williamson S.M., Storey B., Howell S., Harper K.M., Kaplan R.M., Wolstenholme A.J. (2011). Candidate anthelmintic resistance-associated gene expression and sequence polymorphisms in a triple-resistant field isolate of Haemonchus contortus. Mol. Biochem. Parasitol..

[bib43] Yin F., Gasser R.B., Li F., Bao M., Huang W., Zou F., Zhao G., Wang C., Yang X., Zhou Y., Zhao J., Fang R., Hu M. (2013). Genetic variability within and among Haemonchus contortus isolates from goats and sheep in China. Parasites Vectors.

