# Supplementary figures and images for: Using droplet digital PCR for the detection of hco-acr-8b levamisole resistance marker in H. contortus
Source: Int J Parasitol Drugs Drug Resist. 2021 Mar 26;15:168–76. doi: 10.1016/j.ijpddr.2021.03.002 (PMC8044644; doi:10.1016/j.ijpddr.2021.03.002)

S1(ISE)

S2(Weybridge)

S3(Zaire)

Neg-

500

100

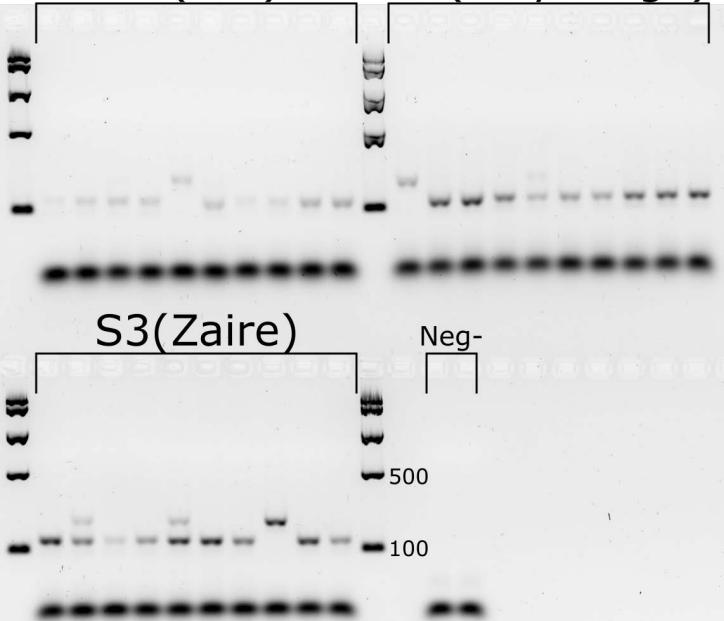

Supplement: Multimedia component 2 [file mmc2.pdf]

R1(Cedara)

R2(Borgsteede)

R3(Kokstad)

Neg-

500

100

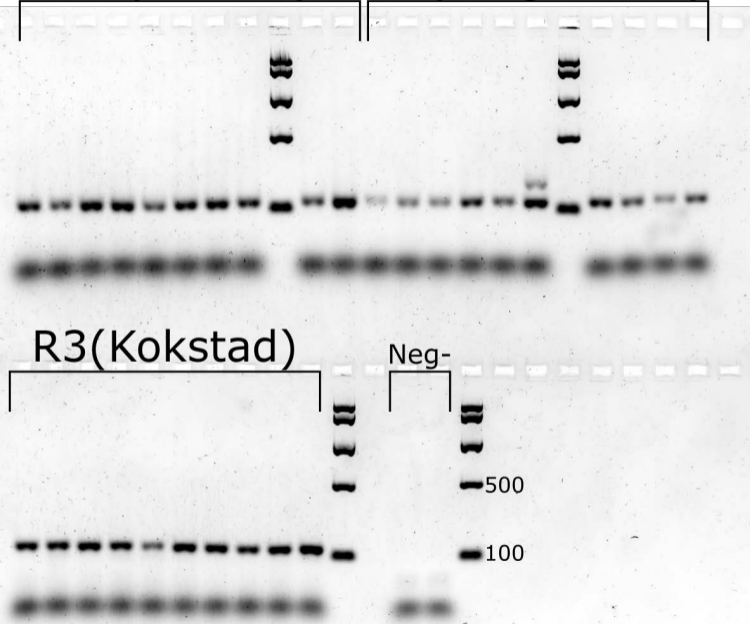

Supplement: Multimedia component 3 [file mmc3.pdf]

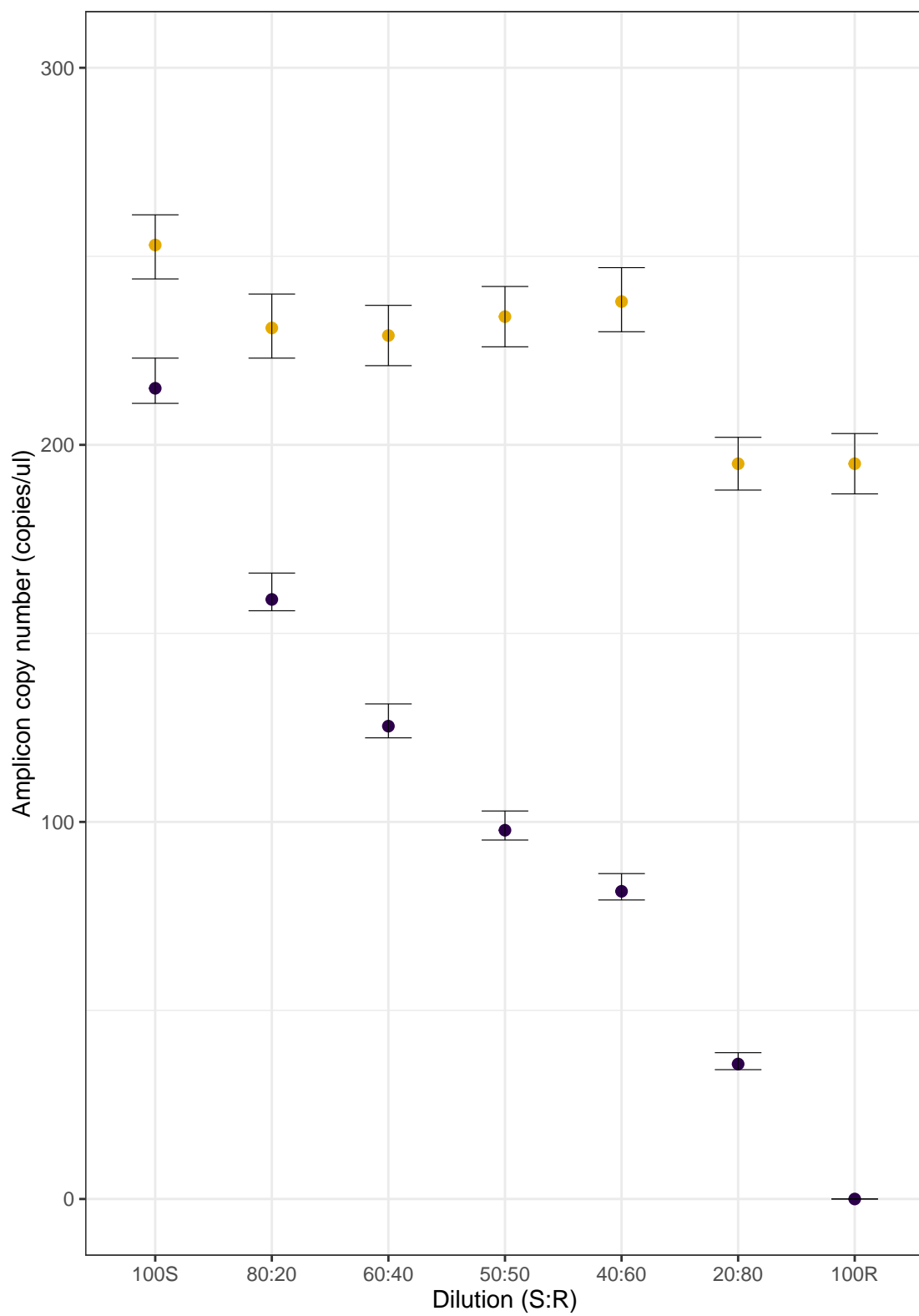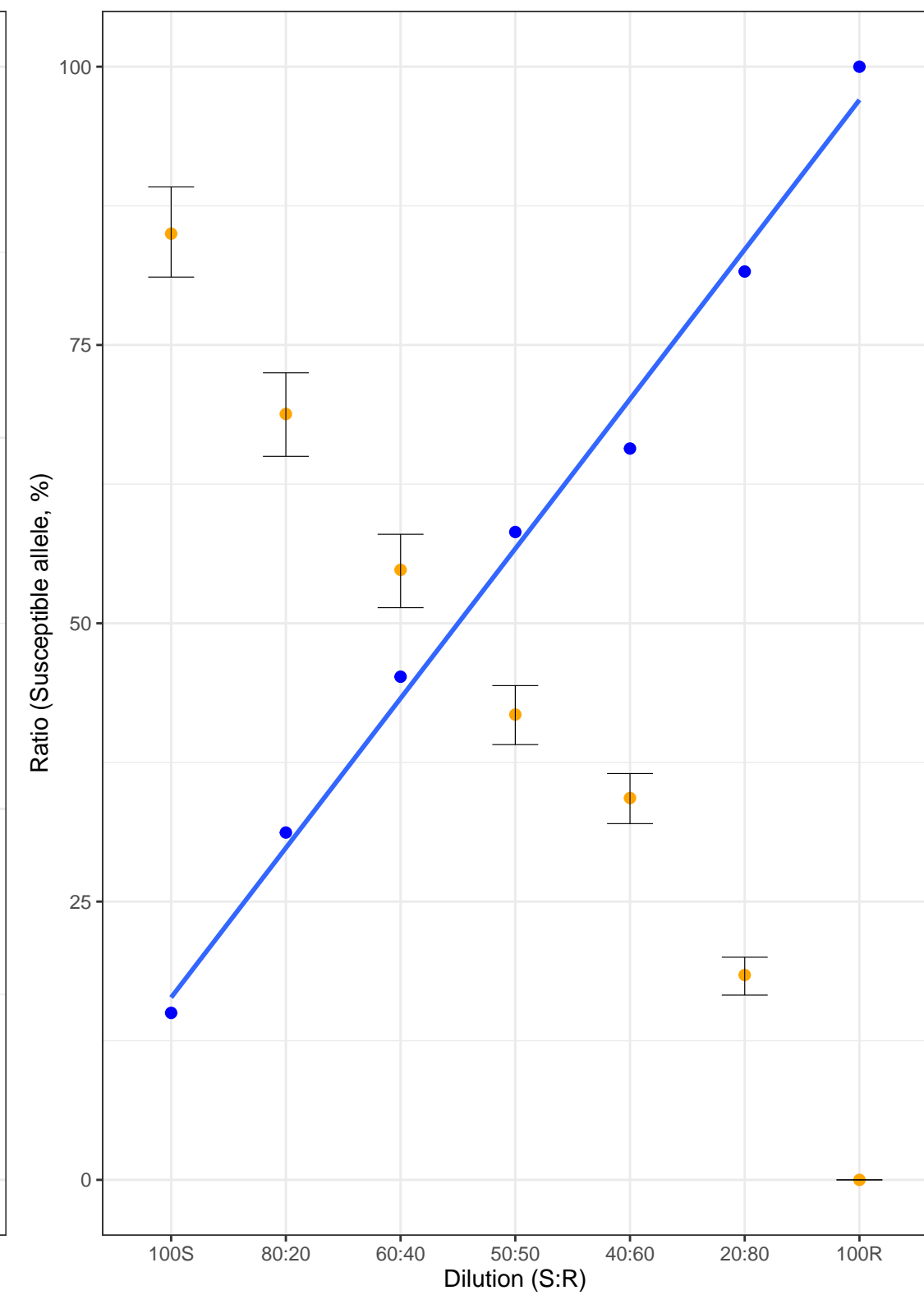

Supplement: Multimedia component 4 [file mmc4.pdf]
