# Supplementary material for: Using droplet digital PCR for the detection of hco-acr-8b levamisole resistance marker in H. contortus
Source: Int J Parasitol Drugs Drug Resist. 2021 Mar 26;15:168–76. doi: 10.1016/j.ijpddr.2021.03.002 (PMC8044644; doi:10.1016/j.ijpddr.2021.03.002)
Supplement: Multimedia component 5 [file mmc5.docx]

|  | **Susceptible isolates** | | | |  | **Resistant isolates** | | | |
| --- | --- | --- | --- | --- | --- | --- | --- | --- | --- |
| **Isolate** | **DNA conc. ng/µl** | **Sequencing** | **PCR** | **ddPCR** | **Isolate** | **DNA conc.**  **ng/µl** | **Sequencing** | **PCR** | **ddPCR** |
| ISE1 | 12,22 | *-* | *resistant* | *heterozygous* | Ced1 | 20,33 | resistant | resistant | resistant |
| ISE2 | 10,90 | resistant | resistant | resistant | Ced2 | 21,01 | resistant | resistant | resistant |
| ISE3 | 11,34 | *-* | *resistant* | *heterozygous* | Ced3 | 15,04 | - | resistant | resistant |
| ISE4 | 10,52 | - | resistant | resistant | Ced4 | 16,83 | - | resistant | resistant |
| ISE5 | 8,76 | *susceptible* | *susceptible* | *heterozygous* | Ced5 | 22,82 | - | resistant | resistant |
| ISE6 | 11,39 | *-* | *resistant* | *heterozygous* | Ced6 | 12,06 | resistant | resistant | resistant |
| ISE7 | 10,43 | *-* | *resistant* | *heterozygous* | Ced7 | 21,25 | resistant | resistant | resistant |
| ISE8 | 11,06 | resistant | resistant | resistant | Ced8 | 21,37 | resistant | resistant | resistant |
| ISE9 | 12,37 | - | resistant | resistant | Ced9 | 11,47 | - | resistant | resistant |
| ISE10 | 12,56 | - | resistant | resistant | Ced10 | 16,09 | - | resistant | resistant |
| W1 | 15,33 | susceptible | susceptible | susceptible | BR1 | 11,62 | resistant | resistant | resistant |
| W2 | 14,76 | resistant | resistant | resistant | BR2 | 12,42 | resistant | resistant | resistant |
| W3 | 15,66 | resistant | resistant | resistant | BR3 | 8,93 | resistant | resistant | resistant |
| W4 | 14,92 | - | resistant | resistant | BR4 | 11,79 | resistant | resistant | resistant |
| W5 | 18,09 | *-* | heterozygous | heterozygous | BR5 | 7,37 | resistant | resistant | resistant |
| W6 | 17,86 | resistant | resistant | resistant | BR6 | 8,82 | *resistant* | *heterozygous* | *heterozygous* |
| W7 | 17,67 | resistant | resistant | resistant | BR7 | 8,75 | resistant | resistant | resistant |
| W8 | 14,23 | resistant | resistant | resistant | BR8 | 7,52 | - | resistant | resistant |
| W9 | 21,13 | - | resistant | resistant | BR9 | 3,2 | - | resistant | resistant |
| W10 | 14,60 | - | resistant | resistant | BR10 | 6,96 | - | resistant | resistant |
| Zai1 | 14,34 | resistant | resistant | resistant | Kok1 | 25,12 | resistant | resistant | resistant |
| Zai2 | 13,23 | *resistant* | *heterozygous* | *heterozygous* | Kok2 | 24,09 | resistant | resistant | resistant |
| Zai3 | 13,71 | resistant | resistant | resistant | Kok3 | 26,19 | - | resistant | resistant |
| Zai4 | 10,74 | resistant | resistant | resistant | Kok4 | 29,25 | resistant | resistant | resistant |
| Zai5 | 10,21 | *resistant* | *heterozygous* | *heterozygous* | Kok5 | 22,79 | resistant | resistant | resistant |
| Zai6 | 11,44 | resistant | resistant | resistant | Kok6 | 22,04 | - | resistant | resistant |
| Zai7 | 12,95 | resistant | resistant | resistant | Kok7 | 13,79 | resistant | resistant | resistant |
| Zai8 | 9,61 | susceptible | susceptible | susceptible | Kok8 | 19,79 | resistant | resistant | resistant |
| Zai9 | 6,85 | - | resistant | resistant | Kok9 | 20,54 | - | resistant | resistant |
| Zai10 | 10,65 | - | resistant | resistant | Kok10 | 12,25 | - | resistant | resistant |
