# Supplementary material for: Using droplet digital PCR for the detection of hco-acr-8b levamisole resistance marker in H. contortus
Source: Int J Parasitol Drugs Drug Resist. 2021 Mar 26;15:168–76. doi: 10.1016/j.ijpddr.2021.03.002 (PMC8044644; doi:10.1016/j.ijpddr.2021.03.002)
Supplement: Multimedia component 1 [file mmc1.pdf]

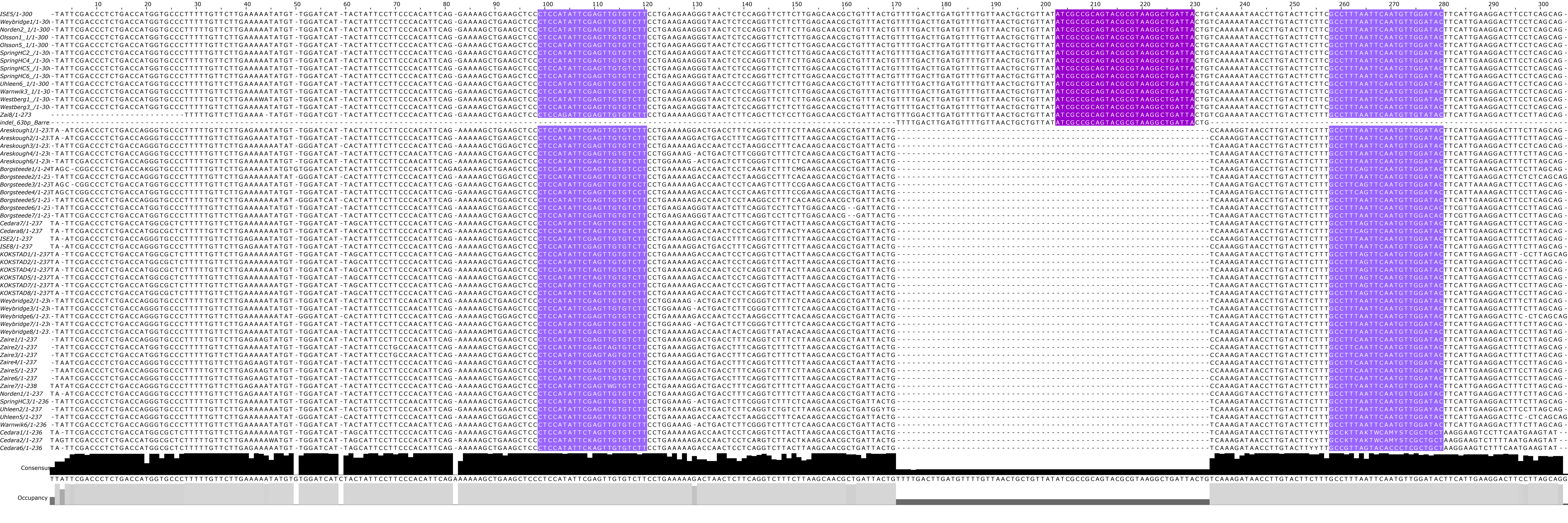
Consensus
Occupancy
